# A Method for Conducting Preliminary Analysis of the Nature and Context of Sport for Development and Peace Projects in Fieldwork Research: An Illustration With a Malagasy Non-Governmental Organization

**DOI:** 10.3389/fspor.2021.658496

**Published:** 2021-11-04

**Authors:** Tegwen Gadais, Laurie Décarpentrie, Andrew Webb, Marie-Belle Ayoub, Mariann Bardocz-Bencsik, Claude Bélanger

**Affiliations:** ^1^Department of Physical Activity Sciences, University of Quebec in Montreal, Montreal, QC, Canada; ^2^UNESCO Chair in Curriculum Development (UCCD), University of Quebec in Montreal, Montreal, QC, Canada; ^3^Department of Psychology, University of Quebec in Montreal, Montreal, QC, Canada; ^4^Sprott School of Business, Carleton University, Ottawa, ON, Canada; ^5^Doctoral School, University of Physical Education, Budapest, Hungary

**Keywords:** sport for development and peace (SDP), non-governmental organizations (NGOs), annual report, Africa, decision making, actantial model

## Abstract

More research on sport for development and peace (SDP) organizations is needed to better understand their actual contributions to the United Nations (UN) Sustainable Development Goals (SDGs). Yet, the unstable, restricted, or even risky contexts in which many non-governmental organizations (NGOs) and SDP agencies sometimes operate often leave researchers to face important challenges to develop effective or feasible methods to work with such organizations. This study aimed to address the ontological and epistemological questions about what should be known about a given context in an organization before setting off on fieldwork. We propose a methodology, based on an actantial model (AM), as a method to analyze the nature and context of a project, to assess the actors involved in the project, and to establish if the global cost (i.e., material, temporal, financial, and physical) for conducting fieldwork is realistic and feasible of all the parties involved in the potential project. To illustrate this process, we analyzed the nature and context of an SDP project in Madagascar as the first step for potential collaborative research. As researchers, we do not want to invest time and energy to build up a fully developed field research project with an NGO in a context where it would not be realistic or feasible to conduct such research. Actually in this context, developing a research protocol without an implementation strategy might not only be detrimental to the researchers, but also to the NGO itself, where resources are often limited. Accordingly, the results from this preliminary field research demonstrate that an AM is a relevant analytical tool for obtaining insights about the context, the actors, and their relationships within an NGO. In conclusion, this model might be a useful instrument for conducting an initial analysis for the preliminary identification of the necessary conditions for the construction of a sustainable empirical research partnership with a given SDP project.

## Introduction

The sport for development and peace (SDP) initiatives are currently flourishing in various contexts and in different areas of the world (Svensson and Woods, 2017). Even so, the questions remain about how to find effective ways to conduct such research with the SDP agencies that are often established in regions of the world that often intervene in unstable or difficult contexts such as climate catastrophes, civil wars, socio-economic or political crises, and extreme poverty. Therefore, it is important to examine the feasibility of given research in an environment where unpredictable events could sometimes make the work of an organization difficult and challenging (Armstrong, 2004; Vlassenroot, 2006; Brück et al., 2015; Koddenbrock, 2015). In addition to the documented challenges of conducting SDP research with marginalized populations, the barriers for conducting such research include intangible obstacles, such as SDP personnel that are hesitant about working with academics (Welty Peachey and Cohen, 2015). Accordingly, it remains important to better understand the issues facing a given SDP project, such as the energy, time, and cost that will be necessary for the research project. In this context, it is effective for the researchers to analyze if it is pertinent to do fieldwork with a given organization before they would start a formal project with them. This *modus operandi* is fruitful in the academic context where limits and constraints are numerous, including the tenure and promotion systems in higher education institutions that encourage quick and regular publications (Welty Peachey and Cohen, 2015).

In spite of the importance of this procedure, only few field researchers have attempted to answer this question (Collison and Marchesseault, 2016; Collison et al., 2016). For instance, Collison et al. (2016) argue that the encultured informants will play a key role in obtaining and maintaining access to a research site. Yet, overall, little information is available about how to identify and activate these essential encultured informants. An actantial model (AM) might be a useful tool to gather basic information to avoid these pitfalls in the analysis of SDP projects operating in unstable contexts.

To be in line with the above-mentioned procedures, and with the objective to better understand the inner functioning of a given NGO, the SDP analysts (e.g., researchers and program evaluators) should (a) gain an understanding of the context in which the NGO operates and (b) identify the encultured informants before launching a full research project. This should be attainable through the analysis of available written materials and documents that are made available by the SDP organizations as a window on their work and performance. However, going through these documents can be tedious and time-consuming, so an efficient method is needed to approach this task. Accordingly, we propose to test an AM of Greimas (Greimas, 1983) as an instrument to reach this objective. This model, inspired by the study of folktales, positions actors in an organization according to their role in what here is called a story. This model has previously been used in management research (Breton, 2009; Gendron and Breton, 2013; Hasbani and Breton, 2013) and in the analysis of SDP program management (Gadais et al., 2017; Webb, 2019). This modelization is promising to generate insight into the story of organizations through their annual report. Even if this method has shown promising results in other areas, scholars still need to establish the limits and potential of the AM if applied to the SDP domain. Thus, an AM could be a powerful tool for informing decisions regarding whether to proceed with given fieldwork or not.

## Reviewing the Literature

### SDP: Projects and Research

The number of SDP projects throughout the world has significantly increased in recent years (Schulenkorf et al., 2016; Svensson and Woods, 2017; Bardócz-Bencsik, 2020). In these projects, the sport is used as a lever for social integration in developing countries, areas affected by conflict, and disenfranchised or underserved locations in the developed countries. SDP has been defined as "the intentional use of sport, physical activity, and play to achieve specific developmental goals in the low-income and middle-income countries and disadvantaged communities in high-income areas,” and includes “all forms of physical activity that contribute to the fitness, mental well-being, and social interaction, such as play, recreation, organized or competitive sport, and indigenous sports and games” (UN Inter-Agency Task Force on Sport for Development and Peace, 2003;^1^ Richards et al., 2013). These definitions have been widely adopted by the SDP actors and researchers (Schulenkorf and Adair, 2014; Webb and Richelieu, 2015).

One explanation for the popularity of SDP lies in the common-sense belief that, in addition to its health benefits, the sport has a number of social advantages. According to Lyras and Welty-Peachey (2011), the sport-based programs use sport as a medium “to exert a positive influence on public health, the socialization of children, youths and adults, the social inclusion of disadvantaged, the economic development of regions and states, and fostering intercultural exchange and conflict resolution.” While the development of SDP theory receives increasing attention from the scholars (Schulenkorf et al., 2016), some authors note that the scientific literature lacks empirical approaches for understanding the mechanisms by which sport can foster the development of participants (Hartmann and Kwauk, 2011; Welty Peachey and Cohen, 2015). A review by Schulenkorf et al. (2016) found that, since 2000, there has been an increasing trend in the focus of publications on the social and educational outcomes related to youth sport, with football (soccer) being the most common activity. In spite of this, empirical research in the SDP field remains underdeveloped when compared with the theoretical advancements and innovations in other aspects of the SDP projects (Lyras and Welty-Peachey, 2011). Moreover, the majority of SDP scholars focused on the community level, where primarily qualitative approaches are used. The geographical contexts of authorship and study location present an interesting pattern: although the majority of SDPs are carried out in Africa, Asia, and Latin America, and 90% of the authors of these studies are based in North America, Europe, and Australia (Schulenkorf et al., 2016). The same tendency is demonstrated by another literature review on SDP (Svensson and Woods, 2017). Globally, empirical research in the SDP field remains underdeveloped when compared with the theoretical advancements and innovations in other aspects of SDP projects (Lyras and Welty-Peachey, 2011).

### Research Methods on SDP

The majority of SDP research has thus far contributed to the conceptualization and development of theoretical perspectives in this field (Gadais, 2019). Conceptual research has established that sport can positively affect a number of outcomes if designed and managed well. Sport can help individuals, increase social capital, and reduce social exclusion (Sherry, 2010; Sherry and Strybosch, 2012; Welty-Peachey et al., 2013), it can enhance social capital in the ethnically divided communities (Schulenkorf et al., 2011), and it can play a vital role in peace-building efforts by helping reduce prejudice (Sugden, 2010; Lyras, 2012; Welty-Peachey et al., 2015). However, more empirical studies are still needed to bridge the gap between the theory and practice (Welty Peachey and Cohen, 2015; Gadais, 2019) and several scholars have highlighted the need to better assess the efficacy of sports to influence development or peace (Chawansky, 2014). As such, theory-building has been limited in this area, from both the theory-to-practice and practice-to-theory perspectives (Lyras and Welty-Peachey, 2011; Schulenkorf, 2012; Coalter, 2013; Edwards, 2015), even if the authors have proposed milestones to begin to fill this gap (Gadais et al., 2021b).

A large number of organizations, such as NGOs contributing to the SDP sector, have their own approaches and agendas with regards to the United Nations' (UN) Sustainable Development Goals (SDGs) for 2015–2030 (United Nations Office on Sport for Development Peace, 2017). Therefore, the relationships between the SDP organizations and the local communities that benefit from these development programs need to be considered. The organizations and communities are two important elements for informing how we might address the call of scholars to bridge the gap between theory and practice and to consider the contextual influences and challenges to theory development (Coalter, 2007, 2013; Schulenkorf and Spaaij, 2015). Choosing appropriate research methodologies remains challenging, as the locations in which SDP agencies operate are frequently hard to reach and difficult to investigate for many context-related reasons (Vlassenroot, 2006; Brück et al., 2015; Koddenbrock, 2015), in environments that are sometimes called extreme development contexts (Gadais et al., 2021a). Our research is targeting those particular SDP projects that have been developed in such contexts.

An additional problem is related to the fact that the NGOs and research projects often work on different timetables and priorities: the NGOs would usually work on a day-to-day basis, while the research projects can take an extended period of time to be implemented and completed. Thus, for the long-range interests of researchers, it is fundamental to determine from the outset of the research whether it is worth investing in and building a partnership with, a given SDP NGO. Moreover, the objective is not to waste the time and energy of NGOs since they often lack human resources, materials, or finances. Thus, a research process may require a great deal of effort on their part to accommodate the research team. The previous research (Gadais et al., 2017) and practical field experience of the authors (Atkinson and Flint, 2001; Abrams, 2010), two humanitarian and development workers and one UN peacekeeper, combined with the previously published academic work, allow us to confidently claim that this type of SDP fieldwork can be difficult and unpredictable and thus is likely to require great investments in both time and resources, from both the research team and the participating organization. Compounding these challenges is the fact that populations targeted by the SDP initiatives are sometimes hard to reach, vulnerable, and living with very complex problems (Armstrong, 2002; Almonte, 2009; Leaning and Guha-Sapir, 2013). Some SDP fieldwork, by extension, could also involve major security issues (Leaning and Guha-Sapir, 2013; Klumpp et al., 2015; Lal and Spence, 2016), as SDP operations frequently take place within unstable environments (Armstrong, 2004; Waxman et al., 2006; Nilsson et al., 2011; Klumpp et al., 2015). For example, the political situations and stability can change rapidly, even during the course of the research; the medical conditions and security for workers can change within a few hours (e.g., climate change catastrophes, such as hurricane or pandemic situations such as coronavirus disease 2019 [COVID-19], Ebola, or swine flu [H1N1 flu]); research agendas can change if disease hits a region where a project is implemented; armed conflicts among many populations with various backgrounds can take place; religion or culture can have unanticipated impacts (Gadais et al., 2021a).

Consequently, the research in the context of a humanitarian crisis involves particularities and idiosyncrasies that are likely to have an impact on the methodological choices of researchers (Ciarli et al., 2010; Brück et al., 2015). According to Vlassenroot (2006), empirical research in conflict situations is subject to many constraints, the most obvious ones are accessibility and security. Despite these constraints, the success of research conducted in the midst of a humanitarian crisis is conditioned less by the degree of insecurity than it is by the ability of researchers to adapt to the changing conditions (Vlassenroot, 2006). This involves the capacity to react rapidly and efficiently to the environment, establish good collaboration with local stakeholders, refine the understanding of the crisis of an individual, and demonstrate methodological flexibility (Atkinson and Flint, 2001). In summary, the researchers need to constantly adapt to the changing reality that is more the rule than the exception in this kind of research, meaning that SDP researchers looking to operate in such contexts still need better tools to help them analyze the risks associated with a given project before hitting the ground. In other words, it is important to ask how researchers can weigh the potential risks and costs against the potential benefits of conducting fieldwork both for the research team as well as the local organization involved. With these considerations in mind, we suggest that there may be new ways of doing this kind of research with the SDP empirical practices. Indeed, several innovative tools and methods have been used in those situations (Ridde and Dagenais, 2012; Brück et al., 2015; Rioux et al., 2018a). One of the promising tools to investigate those specific situations is the AM (Greimas, 1983).

### Actantial Model

**Figure 1:**
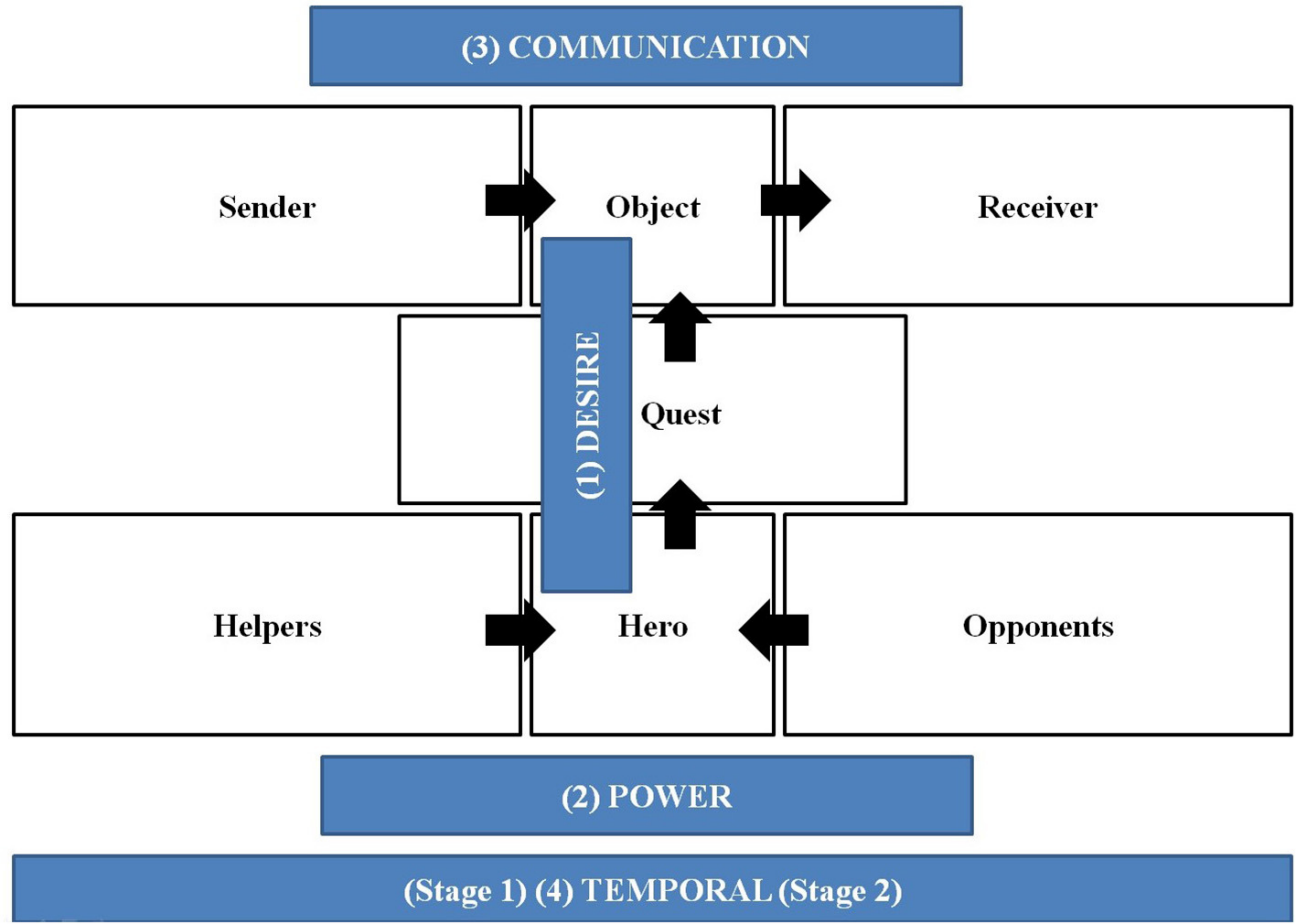
Actantial model and its axis.

An AM by Greimas (Greimas, 1983) is a theoretical model to analyze a project according to its actors, their functions, and their relations. This is usually presented in a report that identifies the actors and their relations and then allocates them to one of six “actant” categories (sender, object, receiver, hero, helper, and opponent). This model, inspired by the study of folktales, positions actors according to their role in a story. Specifically, the *hero* of the story navigates a *quest* to obtain an *object* of value. During the journey, other actors (*helpers*) help the hero reach their goal, while others (*opponents*) try to prevent them from doing so. Additionally, a *sender*, usually for the benefit of a *receiver*, proposes the quest. The quest represents the actions of obtaining the object of value. The actors are assigned to actantial categories according to how they correspond to the roles of functions in the narrative. Several actors can belong to an actantial category (e.g., there might be more than one helper in a story) and the same actor can be found in different categories (e.g., the hero can also be the receiver). An actor is not necessarily a person; it can also be an object, a concept, an event, an element, and more. Also, an actor can be individual or collective and the roles of an actor may shift overtime throughout the narrative. These elements are coded in the terms of a narrative, providing an effective tool for clarifying the relationship between actants for analysis (as shown in Figure 1).

To analyze the structure of relationships of actors within the story, Greimas (1983) identified three axes of relations between all the actors (Figure 1). First, the *axis of will/desire* concerns the relationship established between the hero and the object. Second, an *axis of power* concerns the relationship existing between the hero, the helpers, and the opponents—positive power in the case of helpers and negative in the case of opponents. The helper (e.g., a sword, a horse, or a fairy godmother) aids in facilitating the desired junction between the heroin and the object, while the opponent hinders this junction (e.g., the evil wizard, the dragon, the distant castle, and fear). Third, the *axis of transmission/communication* connects the sender and the receiver. The sender is transmitting an object to the receiver *via* the quest of a hero (for example, the king asks a princess to get a magic wand to free the kingdom).

### Applying the AM to Annual Reports of Organizations

The same categories can be applied to the stories that organizations tell. For instance, the organizations are accountable for their performance and commonly use an annual report to summarize their operations and accomplishments. The annual reports thus represent valuable data for the researchers who want to understand the context. In addition, management scholars have recommended leveraging storytelling as a tool for analyzing the annual reports and documents from organizations (Breton, 2009). For instance, Hasbani and Breton (2013) demonstrated that the AM is a valuable tool when applied to study the annual reports in one company of the pharmaceutical industry. In their study, they demonstrate that this company built a story to explain how this pharmaceutical company (Pfizer, NY, USA) is given a legal mandate to operate by governments (*sender*) to provide health (*object of value*) to the people (*receiver*). Pfizer (*the hero*) accomplished this quest with the help of patents and R&D (*helpers*), which allowed them to develop new products faster than the competition (*opponents*).

Applying the AM in the SDP research field may require certain adjustments and refinements to prepare the fieldwork effectively. Along these lines, this paper proposes that the AM could be used as the first phase of analysis for an SDP organization before the decision is made to develop a complete research protocol with this field partner. One advantage of this approach is that it can be applied to an organization that is distant from the researcher because the AM does not require the researcher to go into the field, as the narrative material is often available online. Performed from a convenient distance for all the parties because it reduces the burden for both the organizations, this method of content analysis facilitates an understanding of the structure of the studied narratives, allowing scholars to appreciate the meta-context of the SDP project, and better understand the roles and functions of each stakeholder. For example, one previous study applied the AM method to an SDP program in El Salvador (Gadais et al., 2017). Through this approach, the AM provided valuable insights about the management priorities and practices within this case. It also revealed that local *Maras* street gangs might make fieldwork hazardous, which is something that needs to be considered before proceeding with fieldwork. Some SDP organizations and projects can be physically hard to reach (Atkinson and Flint, 2001; Abrams, 2010), or are difficult to investigate for other reasons (Almonte, 2009; Leaning and Guha-Sapir, 2013; Klumpp et al., 2015; Lal and Spence, 2016). In addition, more research in SDP in various contexts is needed to bridge the gap between theory and practice (Welty Peachey and Cohen, 2015).

### Objectives of the Present Study

This study builds on Gadais et al. (2017) previous conclusions that using the AM seems to be an effective method of content analysis as applied to the annual reports or other documentation of NGOs (Webb, 2019). By studying the annual reports of an NGO with the AM as a lens, this research aimed to evaluate if (a) a better understanding of the context, the actors, and their relations pertaining to their NGO can be obtained through using the AM, and (b) if these insights can serve to inform decisions about whether or not to proceed with fieldwork. To anchor the analysis on a concrete example, we will study the case of a Malagasy NGO to illustrate our results and conduct the first step of our collaborative work with them. We chose this particular NGO as it is established in an unstable context characterized by frequent climate catastrophes as well as political, sanitary, and/or socio-economic crises, such as extreme poverty.

Specifically, the objective was to answer the two following questions: Are the conditions of this Malagasy NGO^2^ and its context appropriate for implementing an empirical study? In particular, our goals were to identify, describe, and analyze (1) the actors involved in the project and their relations, (2) the object (goals) and the quest (action) of the project, and (3) the evolution of the NGO over 4 years (2013–2016). Through this exercise, we also aimed to look at how annual reports of an NGO can be used as valuable documentation for understanding the situation, context, and state of an organization prior to developing a partnership and working closely with it. The overall objective of the research was thus to generate recommendations on how to proceed before getting involved in a research partnership with an organization and to highlight the advantages and limitations of the AM for understanding the contexts and actants of an NGO.

By applying the AM to annual reports, we intend to (a) operationalize a method for analyzing an NGO and its needs through the project reports, (b) generate data about its environment, and (c) produce insights about the organization that can inform the decision about the pertinence of proceeding with fieldwork for all the parties involved.

## Method

### Research Design

A case study methodology was selected for this research because it is suitable for exploring complex social, managerial, and procedural phenomena when the situation includes many variables, multiple sources of evidence, and broad theoretical propositions that guide the collection and analysis of data (Gee, 2014; Yin, 2014). The three prerequisites by Yin for justifying the use of the case study method are present in this project, specifically: (a) the main research questions are either how or why; (b) the researchers have little or no control over the behavioral events; and (c) the focus of the study is a contemporary phenomenon (Yin, 2014). This study remains descriptive and exploratory and, as such, focuses on describing, in detail, the data collated from the partner NGO, in relation to the context in which the project took place, using the theoretical lens of the AM. We should note that this is one of the first times the AM has been operationalized in the study of international development as a content analysis tool. One previous study of SDP recommended conducting a pre-test with the AM applied to other reports of an NGO (Gadais et al., 2017). This study can be viewed as a direct response to that call for more research.

### Targeted Organization and Annual Reports

*Bel Avenir* (BA) is a Malagasy NGO, founded in 2003, that carries out activities in various fields for young, disadvantaged populations of Madagascar. They are based in two towns, Toliara and Fianarantsoa. Based on their documents, BA is very well established in its local community, organized and operated by and for Malagasy people. For this reason, this local NGO has been less impacted by major events, such as the 2008 international financial crisis. Due mostly to local socio-economic difficulties and climate catastrophes, these regions of south Madagascar—from Toliara, Ifaty, Mangily, and Fianarantsoa—are known to be complex, unstable, and sometimes insecure contexts. The education services of BA are composed of (a) formal education within two schools; (b) non-formal education, such as a sports school and a center for music and arts; and (c) awareness-raising projects, such as international interschool exchanges and the publication of Malagasy stories. Through these services, the organization provides a holistic approach to education for development. The sport activities are proposed specifically to kids living on the street during the strategic hours of the day (e.g., hours without supervision or occupation between the end of the school and going back to their home). Their É*cole de Sport* has been supported by the Real Madrid Foundation since 2012, giving workshops, training, and sports equipment.

This study targets the annual reports of BA published in 2013–2016, available online on their website (http://ongbelavenir.org/). The reports are prepared by Bel Avenir's administrative team. These annual reports are between 25 and 55 pages long in French for stakeholders, and all include three major sections: (1) a general presentation (e.g., the identity of the NGO, sites of the NGO, and partners); (2) activities in the reporting year (e.g., context, basic education program, and education/social inclusion/environment sections); and (3) finances of the reporting year. Each annual report presents: (a) the organization, (b) the context of each year, and (c) the activities conducted during the year. The reports also provide information about the organization; about people involved in the projects, such as members of the board of directors, administration teams, workers, and volunteers involved; about places and locations of the organization (e.g., Toliara and Fianarantsoa); and about the three intervention sections of the organization (i.e., education, social inclusion, and environment).

### Analysis

Following Yin (2014) and Gee (2014), this case and content analysis was conducted in three phases. To deconstruct and reassemble the case study, each report was independently analyzed and then the results were combined: (1) the first task was to read the report and take notes. Two coders, who speak French, were tasked with reviewing and independently coding each element (idea) of the narrative of each report (L'Écuyer, 1990). The coders identified exact quotes and page numbers to facilitate the comparison of their results. In the second task, they identified the actants of the AM (i.e., sender, object, receiver, hero, helper, and opponent) as well as the quests contained in the narrative. Subsequently, they produced a table with each AM category by coding actants and relations. Then, a comparative analysis was conducted to confirm correspondence to the AM based on elements of the narratives. (2) The coders were asked to build the AM for each report by using the elements of the first phase. Specifically for this study, they also built more detailed categories (education, social inclusion, and environment) with the intent of analyzing the three pillars of BA (as shown in **Figure 3**). (3) The coders compared, analyzed, and collated the elements that comprised the AM of each year. They generated a general AM that highlights the actors and their relations and a general story of the BA NGO from 2013 to 2016.

### Trustworthiness in Empirical Data Analysis

In the context of this study, and to ensure methodological rigor, all team members interacted with each other during the different phases of the data analysis (Elo et al., 2014). This co-construction made each researcher a critical partner to the other (Smith and McGannon, 2018). Thus, the three main authors were regularly questioned on the issues relating to the research, data, and results. To reach an intercoder agreement, the interrater reliability technique was used to triangulate data and ensure that results are reliable, reproducible, and consistent (Smith and McGannon, 2018). In concrete terms, the coders, along with other team members, would meet throughout the analysis process to compare and discuss the discrepancies between their analyses to refine the coding so they could proceed with more precise coded data. The goal was not to reach a statistical standard but rather to improve the quality of the coding process.

### Ethical Considerations for Using the AM

The AM can be used in an ethical way. Following the proposals of the authors in management, the AM allows on one hand to obtain a completely transparent and external opinion and, on the other hand, to not disturb the functioning of the partner organization. The extra load of work that the external visitors can represent for an organization has to be considered, acknowledging the limited human and material resources, and the constant crisis they need to face on daily basis. The situation and the context of the organization can be carried out at a distance, without risking conflicts of interest and influence. This provides a great advantage to judge the nature and context of an NGO objectively and adequately. Before analyzing any materials, the contacts had been made with the targeted NGO to discuss the potential of our research regarding their needs and interests. Through a Skype meeting with the board of directors of the NGO (in 2016), the research project and its risks were presented. Because they were willing to improve their services, the NGO was open to the risk of constructive criticism coming from the research project. On the other hand, once this initial external and remote analysis has been carried out, it was possible to verify the results obtained and to discuss with the potential partner to validate its content. This final step in the feasibility analysis was done with the partners in this study is a collaborative approach. Consequently, the partners are aware of and involved in the process of disseminating the results as well as the writing of this study.

## Results

### BA NGO and Its Projects as a Story (Drawing the AM)

**Figure 2:**
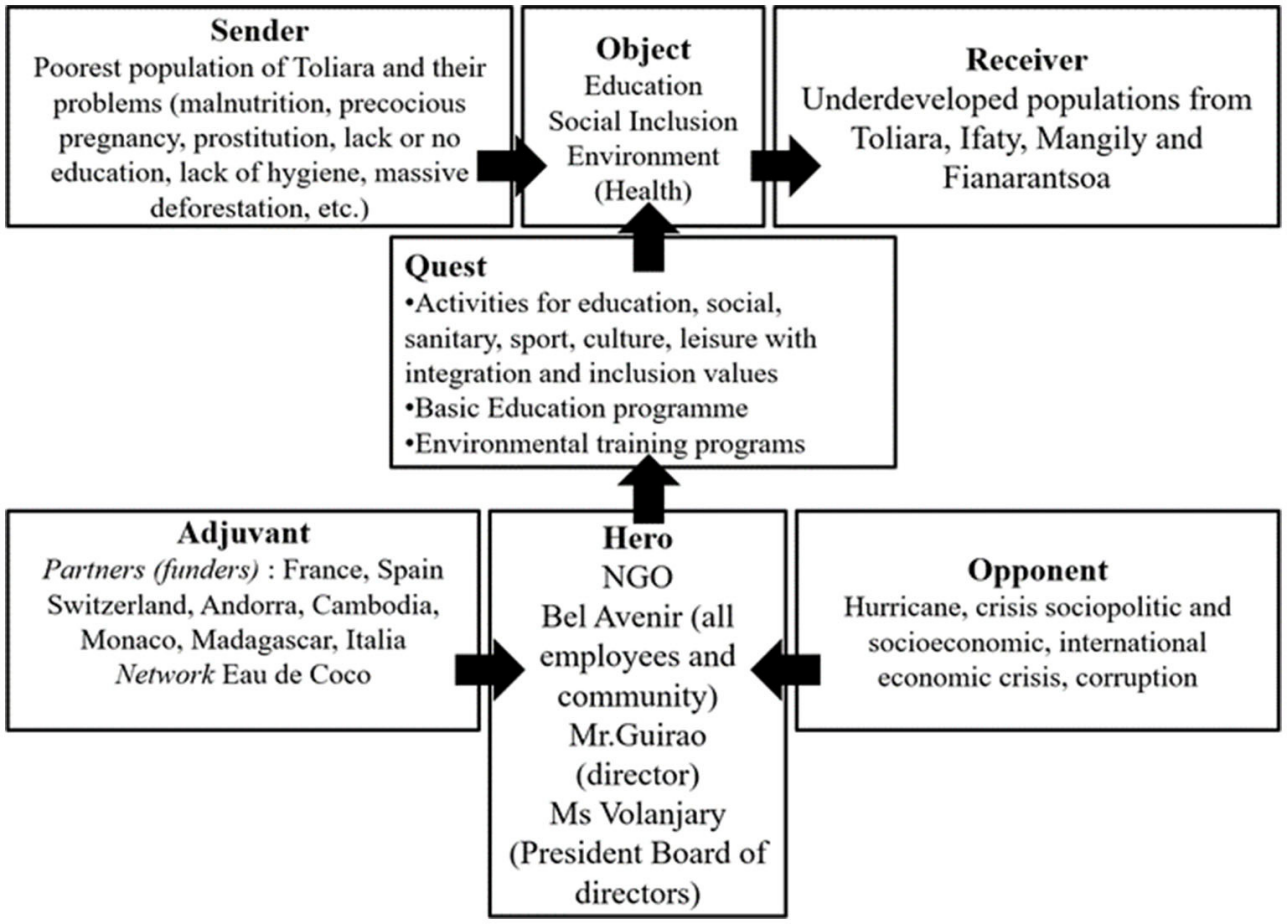
Actantial model of the Bel Avenir NGO and its projets.

Globally over the four reports authored by BA, (as shown in Figure 2) the poorest populations from the towns of Toliara, Ifaty, Mangily, and Fianarantsoa in the south of Madagascar (*senders*) addressed their problems to BA (*hero*). The NGO provided specific activities (*quest*) for education, social support, health, sanitation, sport, music, art, and culture, guided by the values of integration and inclusion. They also offered a basic education program and some environmental training or awareness-raising. BA is presented as a Malagasy organization with a hiring policy that benefits the recipients of the actions of NGOs, such as the local population with a sense of inclusion for people with disabilities. The foreign volunteers complete this local team. Through these projects and actions, BA wishes to reach three main targets (*objects*): education, social inclusion, and environmental protection. During their quest, the administrators and director of BA were helped by the networking of an organization, “Agua de coco,” which has a diversified network established in eight countries; and their funding partners (*helpers*) are mostly from Europe. The heroes had to struggle with various limitations, such as natural catastrophes and disasters (*opponents*), making the context sometimes insecure or inaccessible. Also, the economic crises and socio-politics that brought instability to Madagascar generated very complex situations and made working conditions difficult, partly due to local corruption (*opponents*).

### Actors of BA NGO

Through reading the annual reports of BA, we can understand that the *hero* is the entire NGO, such as all the employees (locals and expatriates), the director, and the president of the board of directors. The *senders* are the most vulnerable among the population in the south of Madagascar (from Toliara, Ifaty, Mangily, and Fianarantsoa). The reports present challenges, such as child labor and a little access to education opportunities (for the educational aspect); early pregnancy, lack of literacy, and/or a lack of hygiene knowledge (for the social inclusion aspect); and massive mangrove deforestation or inappropriate use of agriculture (for the environmental aspect). The *receivers* represent other vulnerable populations, specifically youth and women, from the four mentioned sites where the NGO conducts projects and actions with education as its central aspect.

**Figure 3:**
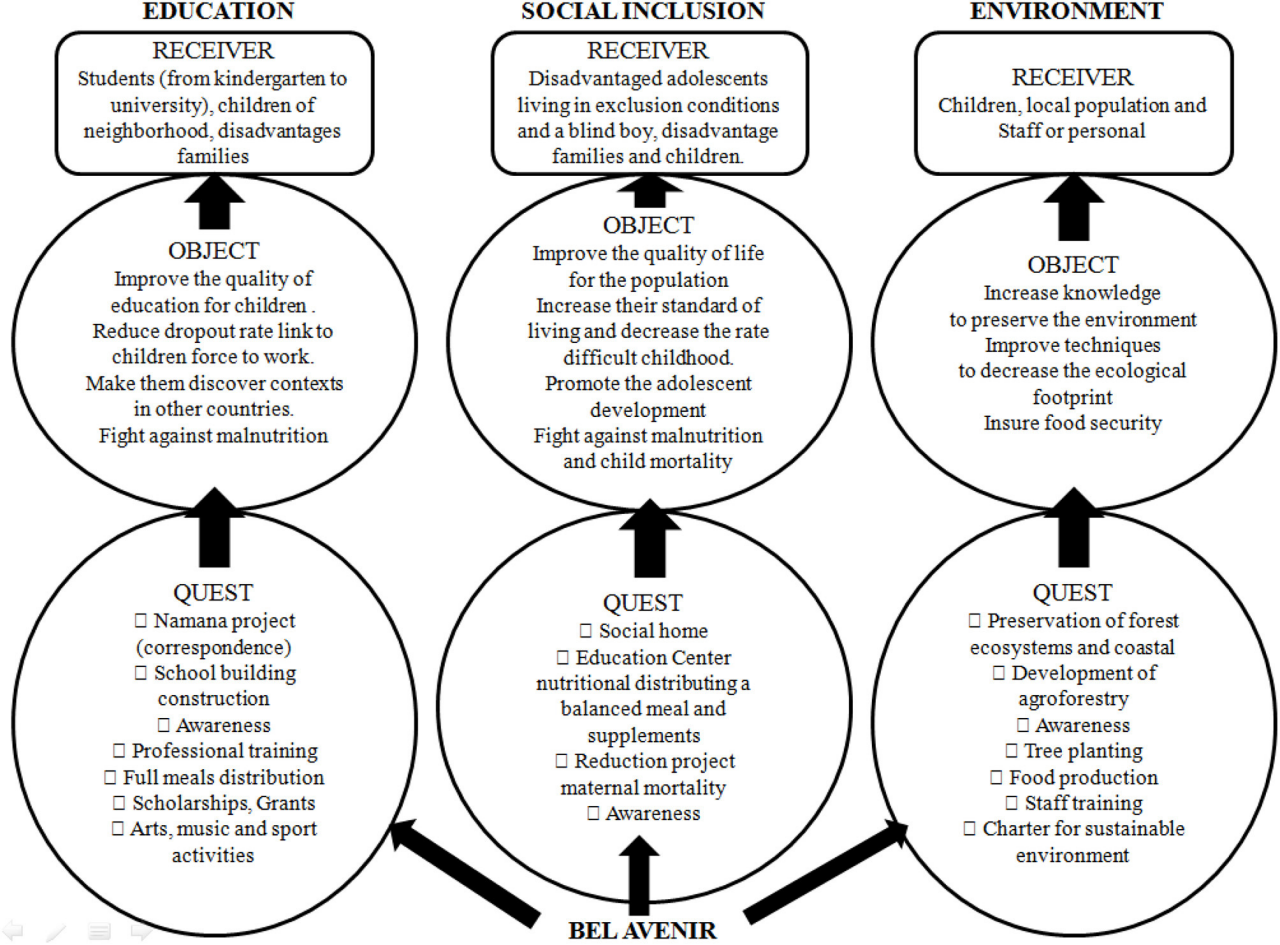
Axis of desire on the three pillars of development in Bel Avenir (Report 2016)^3^.

The BA NGO has three main objects of action: education, social inclusion, and environment (Figure 3). Health was added as a fourth objective action in 2013 and was linked with social inclusion. The projects and actions of BA are organized around the three main pillars. (1) Education: BA provides a basic education program, two private schools, and various awareness-raising activities, literacy, sport, music, arts, and cultural activities inside the *Centre d'art et musique* or the É*cole de Sport*. For example, since 2012, the É*cole de Sport* in Toliara has offered to more than 700 underprivileged children per year the opportunity to educate themselves, have fun, and exercise by participating in sport activities or being part of a football, basketball, or handball team. (2) Social inclusion: the NGO proposes hygiene promotion and nutritional information, for example. (3) Environment: BA manages two agricultural training centers in the towns of Mangily and Fiananrantsoa, and a leisure and environmental awareness center in Mangily.

There are two types of *helpers*. On the one hand, there are external agencies that provide funds to the organization for financing the targeted and specific projects or actions (e.g., the Real Madrid Foundation for the É*cole de Sport*). On the other hand, there is an aforementioned network, named “Agua de coco,” that helps the NGO with the contacts, funding opportunities, skills development, and the like. Finally, *opponents* can be divided into three categories: (1) natural catastrophes, such as hurricane Haruna which struck the region in 2013; (2) political and economic contexts depending on the international events, crises, and the Malagasy government; and (3) corruption, mostly within the local authorities.

### Relations Between Actors of BA NGO

#### The Axis of Desire/Willingness

Three elements are pertinent to highlight in this first axis (Figure 3). First, the hero is a local NGO made by and for the Malagasy population; comprised of locals, it decides which problems need to be addressed first. The position of hero by BA is a concurrent function because this same NGO, which mandates, also provides the activities (quest) for the Malagasy population. Second, the major purpose of BA is to use education as a lever for development. Specifically, BA chooses to address the issues of the Malagasy population by structuring their activities into three main domains of development (i.e., education, social inclusion, and environment,) with a holistic approach. Third, the analysis of the three pillars of BA provides the portrait of real actions and activities of the NGO (as shown in Figure 3, illustration with the 2016 report). For instance, the receivers change depending on which of the three pillars the analysis focuses on. They are the students and disadvantaged families in education; disadvantaged girls, a boy with a visual impairment and disadvantaged families in social inclusion; and the children, villagers/locals, and staff in the environment. Thus, all the objects target several of the UN's SDG, such as no poverty, health and well-being, and quality education.

#### The Axis of Power

The relations with helpers and opponents present several asymmetries because the helper actions do not necessarily balance the challenges generated by an opponent. Various opponents that are usually unpredictable, limit the projects of BA. Hurricanes are an occasional natural catastrophe and no actions by the helpers can eliminate the threat posed by these types of opponents. The socioeconomic and sociopolitical crises, international economic crises, and governmental corruption are not in the control of BA either. The helpers, on the other hand, provide funds, as well as networking or contacts to share the ideas on building skills and competencies, both helpful to realize the projects of NGO.

#### The Axis of Transmission/Communication

This axis connects the sender to the receiver. First, in the case of BA, the senders and receivers are the same people: the most vulnerable among the population of Madagascar. This does not pose a problem in the AM, as the actors are defined not by who they are but by what role they play. The senders addressed BA with the numerous personal and social problems, with the intention of satisfying their needs and improving their quality of life as receivers. The relation is, in this case, self-orientated and circular. Second, the connection between these two actors re-enforces the three pillars of BA: providing education, social inclusion, and environmental services, and activities to the population. From our understanding of the reports, the requests of the sender are well executed and operationalized through the three targets (*objects*) to reach the receiver. These services allowed the population to satisfy their primary needs and helped them improve their quality of life. Third, the reports do not have information on how BA retrieves data on their issues of population, which therefore presents the challenges to evaluate the performance of the organization.

### Evolution of BA NGO From 2013 to 2016 (Axis of Translation)

One of the objectives of the AM is to help the reader understand the story of an organization. Over the 4 years we analyzed (as shown in Table 1), some actants evolved to assume more specific roles. The hero remains the same: BA and the objects maintain the same baseline throughout the 4 years (education, social inclusion, and environment), but have become more specific over the years. For example, from 2013 to 2016, education became the main focus of the activities of BA (*quest*), with secondary programs, such as poverty elimination, capacity building in Madagascar, and sustainable development. In terms of helpers, the organization Agua de Coco Network is the most frequently mentioned, along with the African network for children LAMAKO. The international partners have changed a lot over the years [e.g., World Wildlife Fund (WWF), Orange Foundation, and Covalence Foundation], but some remain constant [e.g., Real Madrid Foundation, Food and Agriculture Organization (FAO), and Enfants du Monde association]. Finally, the opponents were the most changeable and unpredictable parameters over the 4 years. The governmental or police corruption, environmental degradation, natural disasters (e.g., cyclones, hurricane, floods, and drought), an international economic crisis could have affected the funders (in 2008), political crises, and street violence change quickly from 1 year to another.

**Table 1:** Evolution of the NGO over 4 years.

		**1. DESIRE**			**2. POWER**		**3. COMMUNICATION**	
		**Hero**	**Quest**	**Object**	**Helpers**	**Opponents**	**Senders**	**Receiver**
**4. TEMPORAL**	**2013 (T1)**	Bel Avenir	Basic Education programme	EducationSocial Inclusion Environment	Agua de coco African network for children International partners	Environmental degradation Economic crisis Hurricane Haruna Floods Governmental corruption	Poorest populations of Toliara BA staff	Poorest populations of Toliara (children and BA staff)
	**2014**	Bel Avenir	Basic Education programme	Education (main focus) Social Inclusion Environment(Health)	Agua de coco African network for children International partners	Environmental degradation Economic crisis Floods Socio-politic tensions Governmental corruption Street violences	Poorest populations of Toliara BA staff	Poorest populations of Toliara(vulnerable children and adults, BA staff)
	**2015**	Bel Avenir	Basic Education programme	Education (main focus)Social Inclusion Environment(Health)	Agua de coco network African network for children International partners	Drought and Hurricanes Chedza/El Nino Street violences	Poorest populations of Toliara BA staff	Poorest populations of Toliara(vulnerable children and adults, BA staff)
	**2016 (T2)**	Bel Avenir	Basic Education programme	Education (main focus)Social Inclusion Environment(Health)	Agua de coco network African network for children International partners	Drought and Hurricanes Economic crisis Governmental and police corruption	Poorest populations of Toliara BA staff	Poorest populations of Toliara (vulnerable populations)

## Discussion

### The AM in Refining a Research Method for Investigating SDP and for Preparing Fieldwork

The hundreds of organizations implementing programs that contribute to the development and peace through sport are principally NGOs or International NGOs (INGOs) that require support from various agencies, such as sports clubs, federations, national governments, local authorities, intergovernmental organizations, corporations, foundations, and private donors. Their interests are aligned with the 2015–2030 SDGs (United Nations Office on Sport for Development Peace, 2017) and focus on working with marginalized young people. Some of the environments in which they operate can be unstable, insecure, inaccessible, complex, or difficult (Atkinson and Flint, 2001; Armstrong, 2002; Vlassenroot, 2006; Brück et al., 2015). This is because they are often established in the regions of the world that are frequently hit with climate catastrophes, civil wars, socioeconomic or political crises, etc. The researchers, while exploring the limits of what can be known about SDP, would benefit from the tools, methods, or organizational technology (Sandfort, 2010) that adapt to this reality. Overall, proposing and testing a new approach for analyzing the content of SDP reports proved to be a valuable exercise in addition to the previous studies on the topic (Gadais et al., 2017; Webb, 2019). Indeed, this study confirms that the AM remains a promising tool for analyzing the contents of NGO reports and for better understanding the contexts of SDP organizations and their activities. Overall, we submit that it is a good method for helping the researchers decide if they should go forward with a research partnership with an SDP NGO working in an unstable context. Nonetheless, the AM may benefit from adjustments and refinements to produce a more precise picture of the studied network of actors. By applying the AM to four annual reports, we found that valuable insights about management priorities and practices may be obtained through the systematic and rigorous application of this research tool.

### Advantages

Regarding this case study, the AM appears to be a pertinent tool for analyzing an SDP project before proceeding with the complex fieldwork that can be costly in terms of resources for a research team as well as a partner organization. One advantage is that this content analysis tool provides an understanding of the structure of the studied narratives. As noted by a previous study (Gadais et al., 2017), this method allows analysts to appreciate the meta-context of SDP projects, to better understand the roles and functions of each stakeholder (or *actants*), as well as to clarify their relations and the quest itself (*action*). In this sense, the AM allows researchers to consider the contextual influences and challenges to the theory development (Coalter, 2007, 2013; Schulenkorf and Spaaij, 2015; Gadais, 2019), and to build theory by using data from practice (Latour, 2007). As SDP programs can be located in unstable contexts as already mentioned, another advantage of the AM is that it can be applied from a distance, and diminishing the resources load for all the parties. Also, as most annual reports of the NGOs are available online and research data are easily accessible, this method offers the first phase of analysis of an SDP organization and its projects—a research phase that can inform decision-making about pursuing the resource- and time-intensive fieldwork for the research teams and NGOs. The model could act as the initial step for future studies with the NGO because it provides a better understanding of the nature and context of the project. One last advantage is that using the AM on annual reports produced over several years allows the researchers to obtain longitudinal perspectives of the NGOs, thus providing valuable insights into their development through time and the sustainability of the project. Applied to more general reports, this tool could make future fieldwork more effective and efficient because it has the potential to improve the understanding of the researchers of the local context. By extension, this approach of evaluating programs can contribute to revisiting the SDGs 2015–2030 of the UN with a bottom-up perspective (Glaser and Strauss, 1999; Ridde and Dagenais, 2012).

### Limitations

Before investing and requesting from their field partner organization the necessary time and resources to conduct field research, it could be important for the researchers to pre-emptively consider the preliminary portrait of an organization. This involves analyzing a multitude of details and determining the complex relationships constructed through the different elements of the project. Although the AM seems to be a valuable tool for content analysis, one major limitation of this method is that the quality of the information about the context is only as good as the quality of the narratives that are analyzed. The potential role of information in the reproduction of NGO-funder relationships (Ebrahim, 2002) must therefore be considered throughout this decision-making process.

Another limitation to consider is related to the importance of authorship. Undoubtedly, authors of a report must be considered, since the same story, told by a different author, may present the actors in a different light. For instance, a report on the BA occupations would likely be different if the author was an opponent of the NGO. Moreover, considering authorship invites further reflection about the nature and purpose of the analyzed assets. In this case, we purposefully chose to analyze the annual reports, but it is important to remain cognizant of the fact that this form of accounts is intended for consumption by a specific type of reader, which, in this case study, is mostly European. It was beyond the scope of this paper to analyze the different forms of accounts, or to explore the potential discrepancies between formal and informal communication in the non-profit sector. Therefore, it might be possible that the messages intended for the donor base will not provide the rich perspectives researchers need to adequately prepare for fieldwork (Ebrahim, 2002). Our intention was to provide one avenue for preparing for research from a distance. Undoubtedly, the purposefully produced communication assets will not highlight all the major problems or challenges of an NGO. However, they can still provide a pertinent first step in a broader research project that answers whether it is worth conducting the research with an agency before deciding to physically go to its location. In short, the approach proposed here does not give a perfectly clear picture of the context in which the subject NGO operates, but it does provide one basis upon which to decide whether to pursue the partnership with a given NGO.

Finally, concerning the language of the AM, we think that the words used to designate the categories of actants (e.g., sender, receiver, and hero) could be updated so that they use a more appropriate and contemporary language, mindful of the current issues related to power dynamics. This element could be part of the way to improve the use of this model from a decolonizing perspective.

### Better Understand the BA NGO: Lessons Learned Regarding the Pursuance of Fieldwork

The AM provides valuable insights about the evaluation of the conditions needed for sustainable research partnerships with the NGO. First, the BA organization has become robust over time and, despite several crises in its environment, it was still able to produce valuable outcomes. Second, BA takes advantage of its international organizational network. Third, despite an unstable, insecure, complex, and difficult context, BA continues to run its activities by showing flexibility and in the face of adversity, without the appearance of neocolonialist influence (Tar, 2014). New activities and services have been integrated over the examined 4 years, while others have been developed and reinforced. Fourth, over the years, BA has demonstrated various solutions to sustain its programs and actions. Education is the universal driver around which they orient their actions and sport is only one of many tools or activities provided by the NGO. Taken together, these elements argue in favor of a research partnership with BA in the future. The conditions such as the sustainability, robustness, mixed staff, capacity for adaptation, and the holistic approach of the services of BA are favorable for initiating the collaborative research with BA, if we refer to the previous studies in international development (Atkinson and Flint, 2001; Vlassenroot, 2006; Brück et al., 2015). In this sense, this study contributed to the first collaboration with the NGO by providing a tool that is useful in understanding the context and process of the activities and programs of BA.

Through the analysis of SDP perspectives, we understand that the BA É*cole de Sport* was added recently (2012) to the services of the NGO. It is likely that BA used the opportunity offered by the Real Madrid Foundation, recalling what Coalter labels as “just add sports” (Coalter, 2013). However, in this case, BA calls for a holistic approach, to give direction to its programs and activities, and asserts that sport cannot deal with all the problems of beneficiaries (Organisation des Nations Unies, 2010; Wiese et al., 2018). In addition, the É*cole de Sport* provides support to the Toliara public schools for physical education sessions and to improve the quality of education in general. The aim of this program is to provide youth with additional educational opportunities and alternatives to healthy activities by focusing on fair play and keeping them away from risky behaviors. For BA, the É*cole de Sport* is directly linked with the UN SDG 2015–2030: #3 good health and well-being and #4 quality education (United Nations Office on Sport for Development Peace, 2017). However, some questions remain unanswered regarding this SDP initiative: what precisely is the type of SDP intervention that BA provides to youth? What is the impact of sport, music, or art to sustain youth development? Is one of those activities more efficient than the others?

In summary, the AM provides underpinnings for future research efforts and BA succeeded in giving us enough evidence of the stability of their situation and environment. It enables us to investigate further the possibility of developing a research partnership with this NGO. Accordingly, using the AM for preparing the fieldwork has the potential to also contribute to other research methods. These include the co-construction of the intervention between the actors and researchers (Collison and Marchesseault, 2016; Rioux et al., 2018a,b); accidental ethnography, a method for the practitioner-based education research (Levitan et al., 2017); the realistic evaluation program, grounded in the context and circumstances of stakeholders (Ridde and Dagenais, 2012); collaborative research using actors to build scientific knowledge (Desgagné et al., 2001); the Snakes and Ladders model of factors that help or limit the SDP programs (Webb and Richelieu, 2015); and interdisciplinary examples of the studies on SDP (Rioux et al., 2017a,b; Gadais et al., 2021a).

## Conclusion

In an effort to prepare for fieldwork, we described and analyzed the BA NGO from a distance. This NGO works in a context characterized by frequent crisis, as many SDP projects that are established in the regions of the world have unstable contexts due to climate catastrophes, civil wars, socio-economic or political crises, and so on. We applied the AM to four annual reports of BA (2013, 2014, 2015, 2016) using content analysis. Our findings indicate that the AM is a useful tool for analyzing the context of an NGO and for better understanding the actors and their relationships within the NGO. In this case study, the AM was a valuable instrument for the first analysis of an NGO and for beginning to answer whether the conditions exist to construct a sustainable, empirical research partnership beneficial for all the parties investing their resources. This approach helps to articulate the context, the actors involved, and their motivations, and it describes the characteristics of the NGO. Granted, some concepts, such as the role of authorship of the studied reports, still need to be refined to have a clear and complete appraisal of the situation of the NGO. Our application of the AM for analyzing the annual reports of BA highlighted that, in addition to sport, the NGO provides various services, such as music and art activities, and that they extend education and social inclusion to the vulnerable populations of the Toliara and Fianarantsoa region, Madagascar. Our study indicates, through this tool (AM), that the researchers would be justified in considering the fieldwork with BA because of their rich and complex relationships. In other words, the pre-analysis of this NGO using the AM shows that it seems relevant to continue preparing for the fieldwork with this organization.

Hence, this paper proposes a promising research method for collecting the data and improving SDP project implementation when access to the field is complex in regions with unstable contexts; the AM could benefit from being considered by the SDP researchers as an interesting tool for teasing apart the context of an organization by using the annual reports produced by the studied organization. By revisiting this content analysis tool, specifically tailored for SDP research, we have tested a valuable method for operationalizing the content analysis of SDP reports for both the practitioners and scholars concerned with the SDP evaluation. The AM has the potential to provide an understanding of the management of accounts in an SDP context, insights into SDP storytelling, as well as a new way of exploring the SDP landscape through actants and their relationships. Also, the study provides collaborative, partner-oriented research to support the project development of the NGO located in Toliara, Madagascar, *Bel Avenir*. This NGO provides an SDP program at its É*cole de sport* and, consequently, fieldwork with this agency will contribute to the advancement of knowledge in the SDP area.

## Data Availability Statement

Publicly available datasets were analyzed in this study. This data can be found at: https://ongbelavenir.org/qui-sommes-nous/transparence/. We used annual reports of the Belavenir organisation, available online on their web site. They accepted for us to used them as part of the study.

## Author Contributions

TG, LD, and AW were involved in the design of the study and contributed to the review of the literature. M-BA and MB-B conducted the analysis and wrote the results section. TG wrote the first draft of the manuscript, after which LD, AW, M-BA, MB-B, and CB contributed to the revision of the manuscript. All authors have made a substantial, direct and intellectual contribution to the work, and approved it for publication.

## Conflict of Interest

The authors declare that the research was conducted in the absence of any commercial or financial relationships that could be construed as a potential conflict of interest.

## Publisher's Note

All claims expressed in this article are solely those of the authors and do not necessarily represent those of their affiliated organizations, or those of the publisher, the editors and the reviewers. Any product that may be evaluated in this article, or claim that may be made by its manufacturer, is not guaranteed or endorsed by the publisher.

## Footnotes

^1^ https://www.un.org/sport/sites/www.un.org.sport/files/documents/pdfs/SG%27s%20Reports%20to%20GA/A-58-268/A_58_268_ENG.pdf

^2^A research project is currently underway with this NGO and an agreement has been signed with UQAM. This study therefore corresponds to a preliminary work that was initiated in 2018 to prepare in collaboration with the actors of the NGO, the current research projects that address their interests and needs.

^3^ https://ongbelavenir.org/wp-content/uploads/sites/3/2020/01/Rapport-Annuel-2016-Bel-Avenir-Low.pdf
